# Fusion Filters between the No Motion No Integration Technique and Kalman Filter in Noise Optimization on a 6DoF Drone for Orientation Tracking

**DOI:** 10.3390/s23125603

**Published:** 2023-06-15

**Authors:** Minh Long Hoang, Marco Carratù, Vincenzo Paciello, Antonio Pietrosanto

**Affiliations:** 1 Department of Engineering and Architecture, University of Parma, 43124 Parma, PR, Italy; 2 Department of Industrial Engineering, University of Salerno, 84084 Fisciano, SA, Italy; mcarratu@unisa.it (M.C.); vpaciello@unisa.it (V.P.); apietrosanto@unisa.it (A.P.)

**Keywords:** orientation tracking, IMU, MEMS, drone, Kalman filter, no motion no integration filter

## Abstract

The paper works on the new combination between the No Motion No Integration filter (NMNI) and the Kalman Filter (KF) to optimize the conducted vibration for orientation angles during drone operation. The drone’s roll, pitch, and yaw with just accelerometer and gyroscope were analyzed under the noise impact. A 6 Degree of Freedom (DoF) Parrot Mambo drone with Matlab/Simulink package was used to validate the advancements before and after fusing NMNI with KF. The drone propeller motors were controlled at a suitable speed level to keep the drone on the zero-inclination ground for angle error validation. The experiments show that KF alone successfully minimizes the variation for the inclination, but it still needs the NMNI support to enhance the performance in noise deduction, with the error only about 0.02°. In addition, the NMNI algorithm successfully prevents the yaw/heading from gyroscope drifting due to the zero-value integration during no rotation with the maximum error of 0.03°.

## 1. Introduction

**Figure 1:**
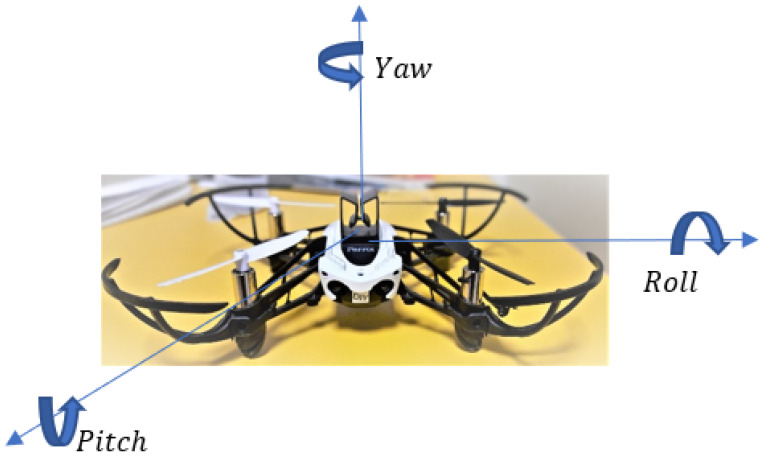
Orientation angles of UAV.

Orientation tracking plays an integral part in the control and stable maintenance of unmanned aerial vehicles (UAV), called drones [1,2]. Nowadays, 6DoF drones have been designed for popular use due to low costs and potential controlling systems. A microelectromechanical systems (MEMS) accelerometer [3,4] and a gyroscope [5,6] are two main sensors used to provide the spatial angles for the drone system. Moreover, the IMU filters are required to optimize the angle measurements due to significant noise from the engine vibration and external interference [7,8]. As described in [9], roll, pitch, and yaw are the Euler angles used for orientation tracking: rotation around the front-to-back axis is called roll, rotation around the side-to-side axis is called pitch, and rotation around the vertical axis is called yaw (Figure 1).

This research focuses on the fusion filters of KF [10,11,12,13,14,15], and NMNI is applied to denoise the oriental signals for the in-depth analysis of the drone vibration impact. A Parrot Mambo drone [16] was used and the data were acquired via the internal IMU sensor inside the drone for the controller system. The noise in the acceleration and angular velocity can be both linear and nonlinear, depending on the manufactured design of the IMU platform [17,18,19,20] and external interference such as engine vibrations. KF combines measurements from orientation sensors with a mathematical model to predict the concerned value. However, KF assumes that everything is linear, and Gaussian can only deal with a unimodal distribution, which can cause the issue of having to deal with the variable drift from the gyroscope. 

Hence, the No Motion No Integration (NMNI) [21] algorithm is utilized to bring the best characteristics out of the gyroscope. In article [22], the NMNI filter was combined with the Madgwick and Mahony [23] filter to minimize the conducted noise in the orientation angles. The research shows that the NMNI is able to enhance the quality of the acquisition signal with less fluctuation and higher accuracy. Its idea is to prevent any single portion of drift from accumulating on the tracking angles by generating a threshold as the boundary between the static and dynamic state. Since the drone does not make the rotation, all the angular velocities will have zero as the ideal state. As a result, the fusion filters between the KF and NMNI can bring out the optimal solution for noise elimination. Moreover, these articles usually mention the extra tools for the experiments, such as GPS, radar, or antenna, which increase the cost and complexity of the system. The Kalman techniques are usually compared via simulation of the position in the *x* and *y*-axis, not mainly on the angle. The research on Euler angles for 6 Degree of Freedom (DoF) drones is still limited. 

In addition, the yaw is the most challenging parameter among Euler angles because the acceleration data on the *Z*-axis remain constant when the drone rotates to the right/left on the Earth frame. This requires additional devices such as the GPS and magnetometer. Without these sensors, the heading value suffers drift from the gyroscope during the integration process. Therefore, the NMNI is applied to narrow the drift and maintain the system stability. From that, the efficiency of the angular velocity integration for the yaw value will be maximized. This article works based on accelerations and angular velocities only to explore the optimized filter in the orientation tracking for the practical drone. The result demonstrates that the NMNI contributes to strengthen the noise filtering capability of KF significantly with less variation during propeller running. The signals are maintained at a stable state without sudden spikes or transients. 

The main contribution can be summarized as follows:

- The detailed explanation of Euler angles in drone systems is demonstrated.
- With the fusion between KF and NMNI, the inclination angles of roll and pitch are significantly upgraded with minimized noise.
- NMNI is utilized to optimize the gyroscope data for the yaw/heading tracking for drones without the support of a magnetometer, GPS, or other devices. This feature reduces the drone system’s complexity and design cost from the large number of sensors.

The article is organized as follows: the first part describes Euler angles in the control system and the KF principle. The next part shows the NMNI algorithms and the threshold update model. Then, the fusion filters between KF and NMNI are analyzed. Finally, the test bench and results analysis are reported. 

## 2. Related Works

In the paper [24], the Complementary filter and Kalman filter (KF) were examined by mounting MPU 6050 [25] on a drone. The result showed higher stability from KF, which had a good command of tracking around the reference point without a significant trend of drift. There are variations in Kalman types, such as Extended Kalman Filter (EKF) or Unscented Kalman Filter (UKF) [26,27,28,29]. However, the EKF must compute Jacobians (first-order derivatives) for process and observation models. If the estimation error on the last step or the additive noises are too large, the EKF may give unsatisfied results. In addition, EKF cannot be used in a system with a discontinuous model since the system is not differentiable because the Jacobian does not exist.

On the other hand, the UKF requires more computational mathematics due to sigma point generation, which causes difficulty during implementation. Accordingly, further support is required to process the angular velocities before feeding into the sensor filters. Therefore, the support of an additional filter to reduce the complicated variation in the gyroscope drift is a good solution to enhance the KF performance. In this research, KF was selected due to its advantages of fast tracking and less complicated implementation, which make it highly suitable to collaborate with the NMNI filter.

To minimize the drift, an article describes the technique of Zero Velocity Update (ZUPT) [30], which applies the zero-velocity condition to static cases. This approach is utilized in indoor navigation, usually applied in Pedestrian Navigation. Even though ZUPT can bind the drift effect, there are various problems in the detection between stationary or dynamic states. The ZUPT execution period is chosen manually based on the necessary level of positioning error increase. Consequently, it is less flexible, which can disadvantage automation. The NMNI algorithm can eliminate the drift accumulation from the beginning to the end of the system operation without specific period setting. The fusion between KF filter and the NMNI has a strong potential to optimize the orientation tracking under noise circumstances, especially the vibration factor. 

Furthermore, yaw angle usually requires the support of a magnetometer or GPS to maintain acceptable accuracy. The accelerometer value does not change during *Z*-axis rotation when the sensor frame is aligned with the Earth frame, and the gyroscope conducts considerable drift over time. Although the magnetometer can track yaw, it needs the robust calibration for iron distortion that grows the complexity of the system and requires the assistance of the GPS [31,32,33] to enhance the precision. In addition, GPS works less effectively in the indoor environment, so accuracy is not guaranteed in this situation. Thus, this article applies the NMNI filter to optimize the gyroscope data for the yaw/heading performance in drones. 

## 3. Materials and Methods

### 3.1. Euler Angles’ Roles in the Controller System

**Figure 2:**
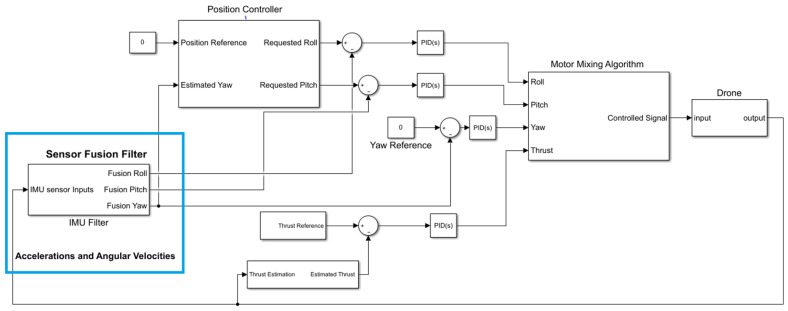
IMU filter in controller system.

During the drone propeller operation, external impacts, such as engine vibration and air density, can induce considerable noise to roll or pitch. The considerable noise also adjusts the altitude and creates some horizontal motion. Consequently, the drone moves away from the reference position. Thus, the IMU filter must be robust enough to remove the noise from the orientation angles to maintain roll and pitch angles properly. As shown in Figure 2, to hover the drone the position controllers take the position reference (X, Y coordinate) as inputs, then output roll and pitch angles, which are the reference inclinations. 

**Figure 3:**
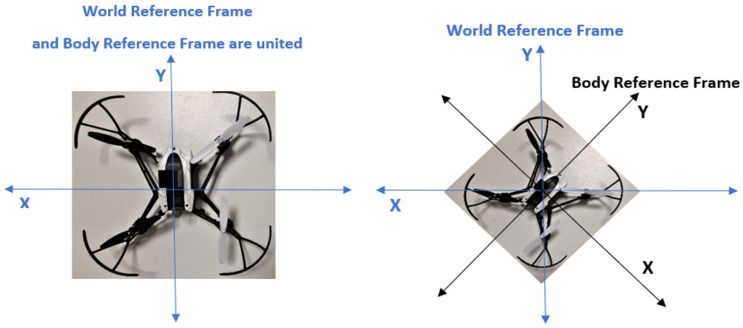
World and body reference frames in two cases.

In this way, the position controller is an outer loop, generating the reference commands for the inner loop of roll and pitch controllers. These are cascaded loops used for controlling the drone system via the PID algorithm. In addition, the measured yaw angle also feeds into the position controller because the XY position error is relative to the world ground, called the world reference frame. Roll and pitch are the inclination of the drone body. Pitch moves the drone in the Y coordinate, and roll moves the drone in the X coordinate; see Figure 3. 

When the drone does not move in the X world direction and Y world direction, it depends on how the drone is rotated, which is measured by yaw. Therefore, yaw is also a crucial parameter to determine whether roll, pitch, or combinations are necessary to achieve the desired point. The position controller uses yaw to convert between the world and body of XY frames. The mixing motor algorithm (MMA) regulates the motor speed for drone moves to keep the system in balance based on the oriental angles. 

Therefore, the roll, pitch, and yaw estimates must be highly accurate and less noisy because they are feedback signals to the controller. With more significant noise, more controller systems have to work for the adjustment, leading to overheating and extensive battery consumption.

### 3.2. Kalman Filter

**Figure 4:**
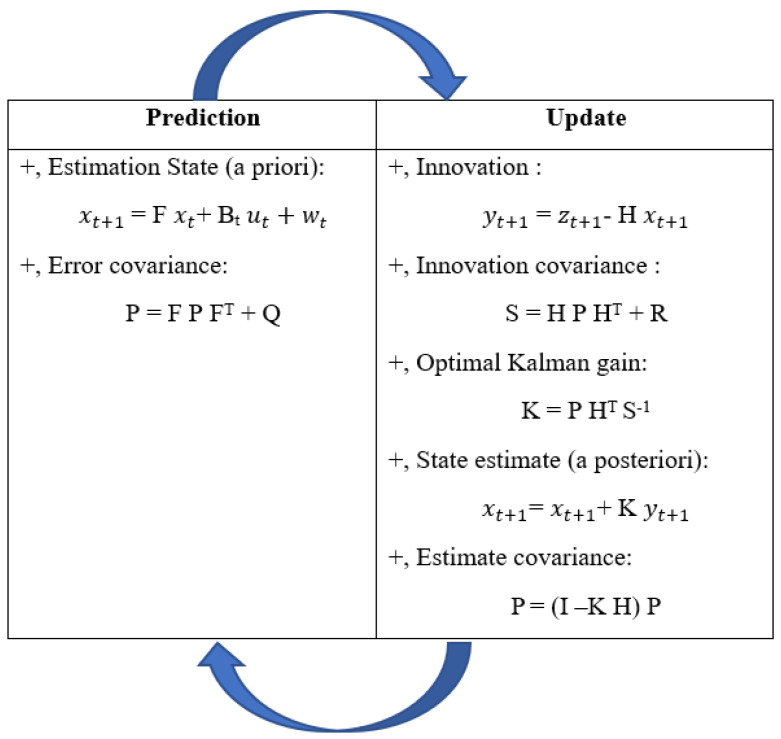
Kalman working principle.

The KF combines input data to provide better results based on uncertain measurement and control system models [34]. The accelerations and angular velocities enter the KF and obtain the denoised orientation angle as outputs. Generally, KF includes two main procedures; prediction and update, as described in Figure 4. The following equations depict the KF principle:

(1) $$x_{t+1}=F\;x_{t}+B_{t}\;u_{t}+w_{t}$$

(2) $$\;\;\;\;z_{t+1}=\;H\;x_{t+1}+v_{t+1}$$

where:

- $x_{t},\;x_{t+1}$ represent the system state vectors at time *t* and *t* + 1, respectively;
- $u_{t}$ is the input vector at time *t*;
- $z_{t+1}$ is the observation (or measurement) at time *t* + 1;
- F is the state transition model, which relates the current states to the next states;
- B_t_ is the control input model, which is applied to the control vector *u_t_*;
- H is the observation model, which maps the true state space in the observed space;
- *w_t_* is the state transition noise, which is attained by a zero-mean normal distribution with the process noise covariance matrix Q[k] where *w_t_* ≈ N (0, Q).

(3) $$Q\left[k\right]=\left[\begin{array}{cc} Qacc & -\Delta T\\ 0 & Q\;bias \end{array}\right]\;$$

This matrix is composed of the estimated state from the accelerometer variance *Qacc* and the variance of bias *Qbias* multiplied by the time interval Δ*T*.

Like *w_t_*_,_ with R as the measurements’ variance, *v_t_* is the noise measurement *v_t_* ≈ N (0, R). The predicted angle for the next loop is the fusion angle from the Kalman filter, just obtained at the current time.

The disadvantages of the Kalman filter are assuming everything is linear, and that Gaussian can only deal with a unimodal distribution, which can be optimized by combining with the NMNI filter. 

### 3.3. “NMNI” Model

#### 3.3.1. Working Principle

**Figure 5:**
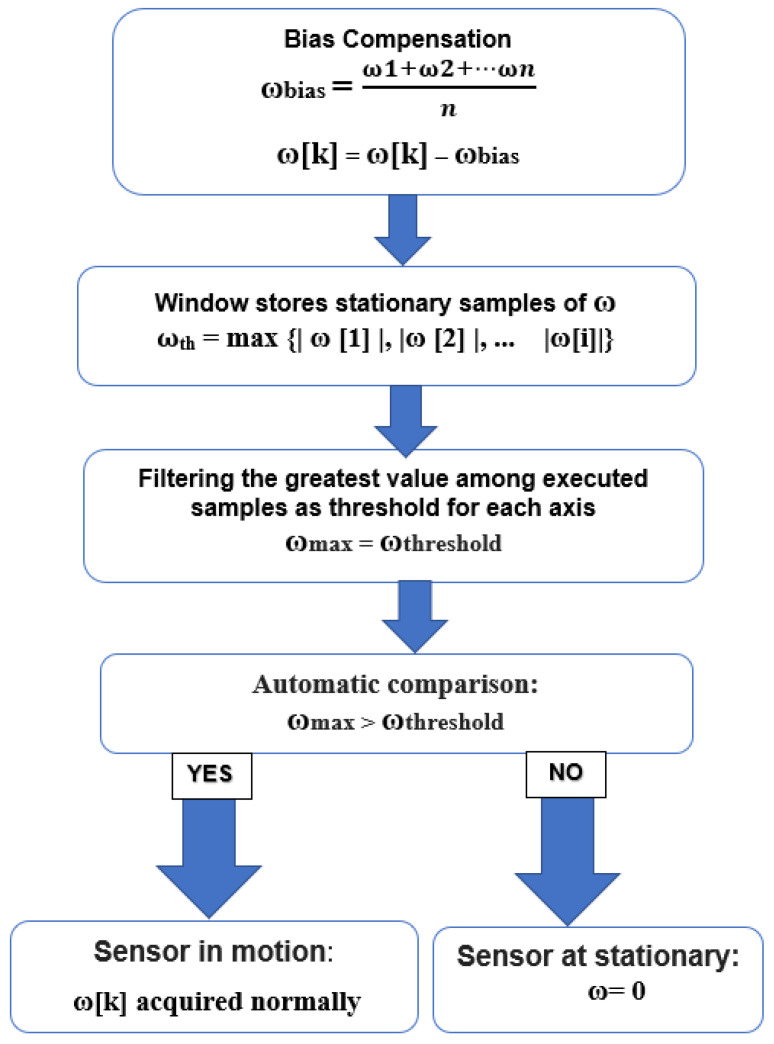
NMNI working chart.

Usually, the yaw value, calculated from gyroscope data, suffers a significant drift from the *Z*-axis (ω). The NMNI system prevents the yaw angle from the integration rate at a static state and only allows the integration process to continue when the subsequent motion occurs. Therefore, the NMNI system uses a threshold to distinguish whether the sensor is in rotation as shown in Figure 5. 

During the start-up phase, supposing no motion, an array of a given number of samples is composed of the absolute value of ω_Z_ with the index of the sample as i (i is an integer number corresponding to the window size, i = 1,…,), and then the filter will keep a ω_th_ with the highest value as the first threshold.

ω_th_ = max { |ω [1]|,| ω [2],… | ω |[i]} (4)

where ω_th_ is the first threshold as the boundary between stationary and dynamic conditions. 

A filter selects the maximum data as ω_th_, becoming the boundary between static and dynamic circumstances. The principle works on the comparison between real-time measured ω[k] and the threshold value ω_Zth_.

|ω [k]|> ω_th_ (5)

→ sensor is in the dynamic case;
→ ω = ω[k].

|ω [k]| ≤ ω_th_ (6)

→ sensor is in the static case;
→ ω = 0.

At this point, the challenge is the variation of gyroscope characteristics due to external factors such as thermal change or vibration, which can be improved by threshold update. 

#### 3.3.2. Threshold Update

**Figure 6:**
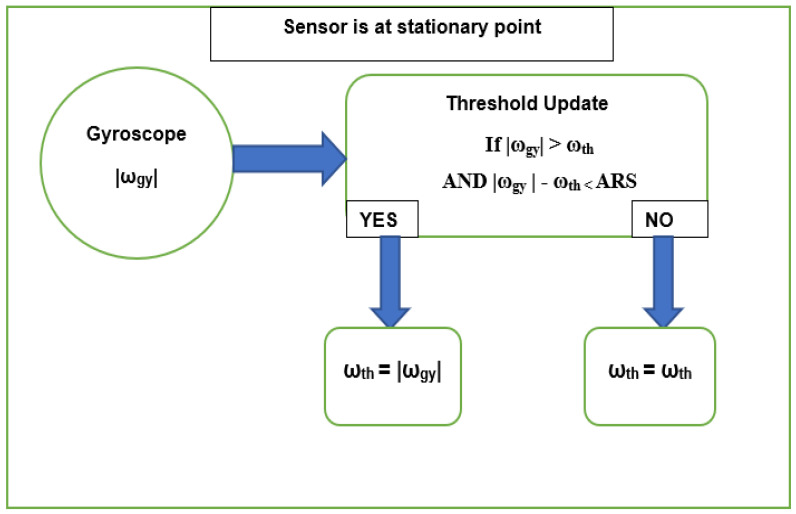
Threshold Update Diagram.

When the sensor is in a stationary position, the angular rate ω_th_ can be updated as demonstrated in Figure 6. When the current ω [k] has an absolute value (abs) higher than the current threshold, these data replace the previous threshold. In this way, the threshold is updated to maintain long-term performance. Furthermore, the threshold is only updated in the case where the difference between the abs of the angular rate and the threshold is less than angular rate sensitivity (ARS), depending on the gyroscope datasheet. Each rotation is relative to the least significant bit (LSB) variation and 1 LSB ≈ ARS.

At the stationary point,

If |ω [k]| > ω_th_ AND |ω [k]| − ω_th_ < ARS (7)

→ ω_th_ = |ω [k]|

For the *Z*-axis rotation, ω_Z_ [k] and ω_,th_ are in the positions of ω_gy_ [k] and ω_th_, respectively. At this point, the drift elimination provides a higher accuracy of the integration outcome. 

### 3.4. Fusion Filters between NMNI and KF

**Figure 7:**
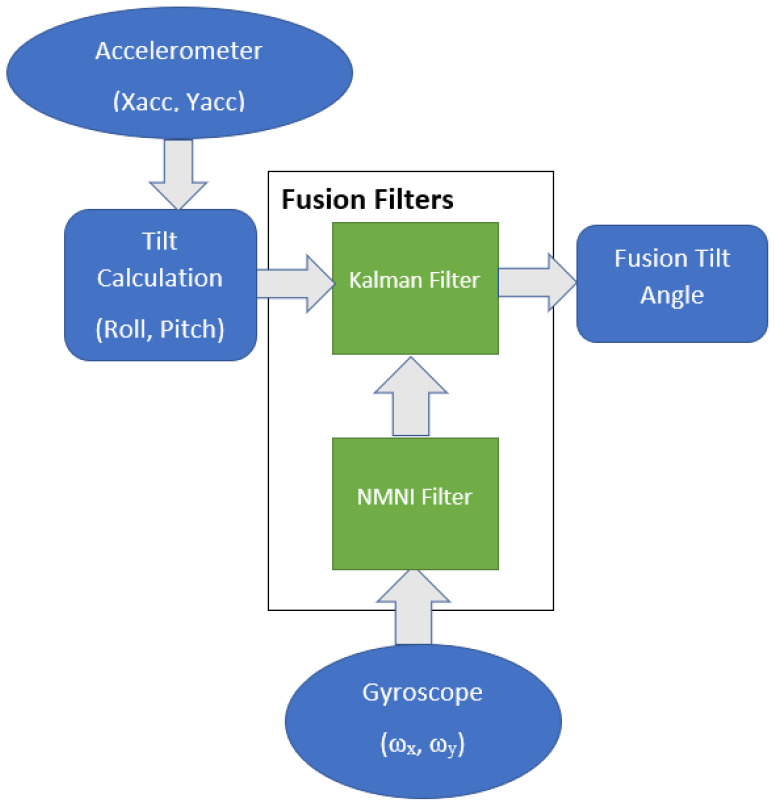
Fusion between KF and NMNI filters for roll and pitch.

The NMNI technique has the essential role of denoising the angular velocities from the gyroscope before entering the KF. For the inclinations, the KF combines the accelerations (Xacc, Yacc) and angular velocities (ω_x_, ω_y_). The roll and pitch are calculated by only the accelerometer via the sine and cosine formula, as described in [9]. The calculated angles are fused with NMNI angular velocities to provide the fusion angle for feedback states as shown in Figure 7. 

### 3.5. NMNI on Yaw Estimation

Due to measurement noise and drift problems, ω_Z_ will vary considerably when the gyroscope rests. After a specified operation time, the obtained results drift away from the range of real angle estimation. In a bad case, the computed result can drift down about 50 degrees after 30 s [35].

(8) $$\mathrm{Yaw}=\;\int_{t_{i}}^{t_{f}}\omega_{Z}\Delta t$$

where *t_i_* is the initial time of the heading rotation, *t_f_* is the stopped time and Δ*t* is the time loop. 

**Figure 8:**
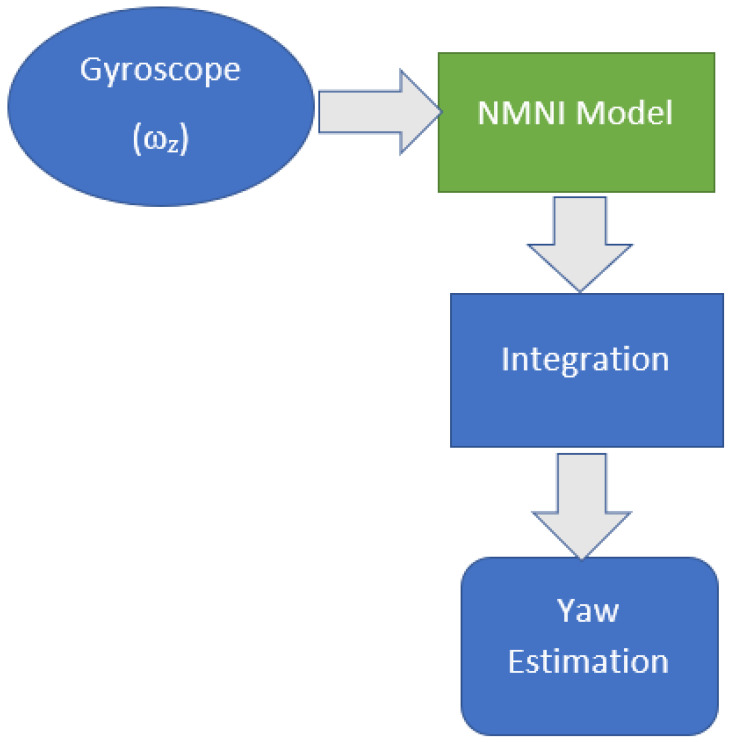
NMNI technique on yaw measurement.

Hence, the NMNI filter is utilized to minimize the drift during the angular integration process. From that, the yaw angle will be optimized as illustrated in Figure 8. The NMNI technique is applied to the angular velocity of the *Z*-axis to minimize the drift accumulated into the integrated angle. The NMNI filter receives noisy ω_z_ and outputs the optimized value for the heading calculation.

## 4. Results

### Experimental Setup

In the experiment, a Parrot Mambo Minidrone was the device under testing for attitude estimation, including 6DOF (3-axis accelerometer and 3-axis gyroscope). The bandwidth was about 100 Hz. The raw accelerations passed through a low-pass filter before entering other sensor fusion filters. 

The Kalman uncertainties were selected based on the following criteria:

- If the estimation is slow, *Qacc* should be decreased to make the algorithm more responsive;
- *Qbias* is supposed to increase when the estimated angle starts to drift;
- If the measurement noise variance R is too high, the filter will respond slowly because the weight on the new measurements is too small. However, if R is too small the value might overshoot and be noisy, since the algorithm gives higher weight to the accelerometer measurements.

After multiple tests, the optimal Kalman uncertainties are selected:

- *Qacc* = 0.004, *Qbias* = 0.05 and R = 0.0005.

The handled mini drone was deployed for real-time control algorithms and sensor fusion methods. The generated MATLAB/Simulink codes were deployed wirelessly to a Parrot Mambo Minidrone over Bluetooth via an external CSR 4.0 Bluetooth adapter to connect the host computer and the Parrot Mambo drone [36]. The Simulink support package for the mini parrot drone was installed and set up for the Parrot Mambo minidrone as outlined in [37]. The drone was controlled using Matlab/Simulink, based on the PID model. The advanced process and result are demonstrated via the performance validation before and after the NMNI fusion filter. 

**Figure 9:**
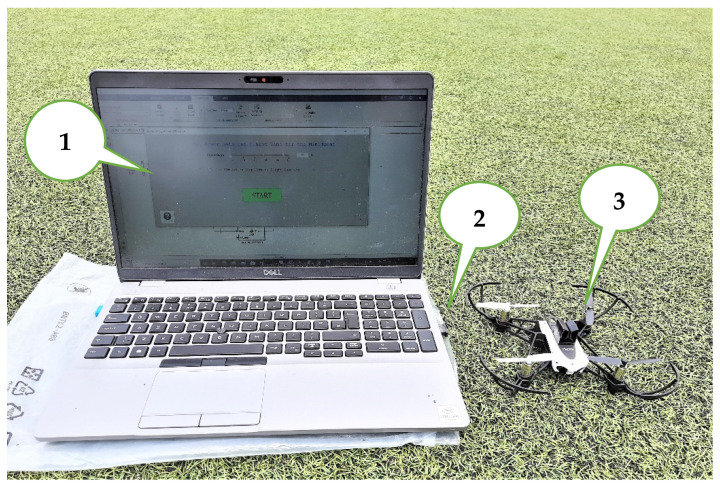
Test bench in the outdoor environment.

The drone test setup includes three main components, as shown in Figure 9.

1. The PC with a MATLAB/Simulink support package for the mini drone;
2. External CSR 4.0 Bluetooth adapter;
3. Parrot Mambo drone.

## 5. Result and Analysis

**Figure 10:**
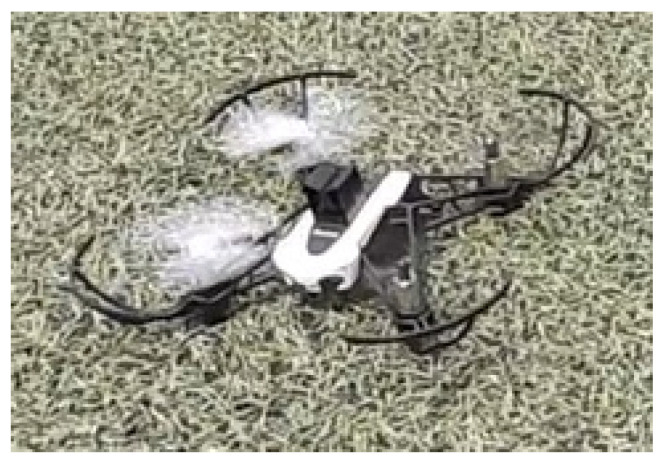
Drone in the outdoor space.

The main goal of the experiment was to analyze the behavior of the IMU filters before and after utilizing NMNI on KF. The drone propellers were controlled to rotate at enough speed to keep the drone on the ground. This way, the drone was in a balanced state, where the actual roll and pitch angles were zero but it still suffered vibrations from the motor operation. The drone was placed on the zero-inclination place measured by the Sun company’s avalanche slope meter [38], as shown in Figure 10. 

### 5.1. Inclination Estimation

**Figure 11:**
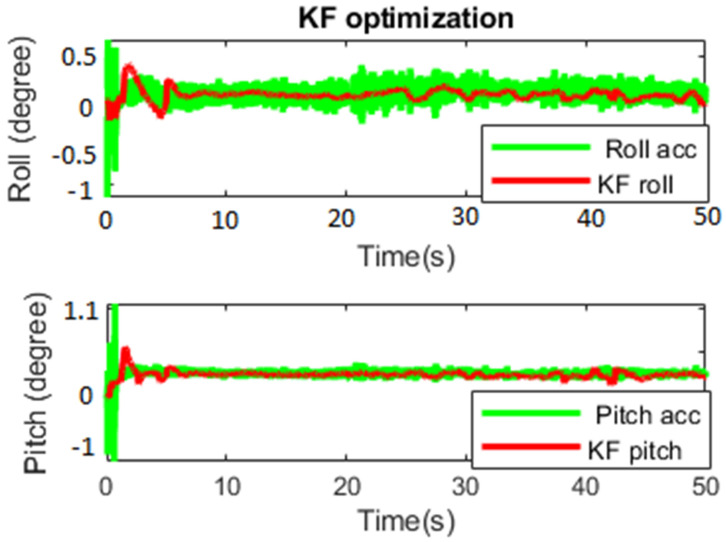
Noise minimization by KF.

The first test was carried out to see the KF capability of noise deduction for 50 s. Figure 11 shows the performance of KF and the normal inclination estimation from only using an accelerometer (Roll_acc and Pitch_acc). KF successfully decreased the variation in the oriental estimation from Roll_acc and Pitch_acc. The pitch suffered more noise from the engine vibration, with a more significant transient and larger variation. 

The starting transient and mean value became smaller after applying KF in both roll and pitch, as shown in Table 1. The KF used prediction and updates to achieve better behavior of the measured data. 

**Figure 12:**
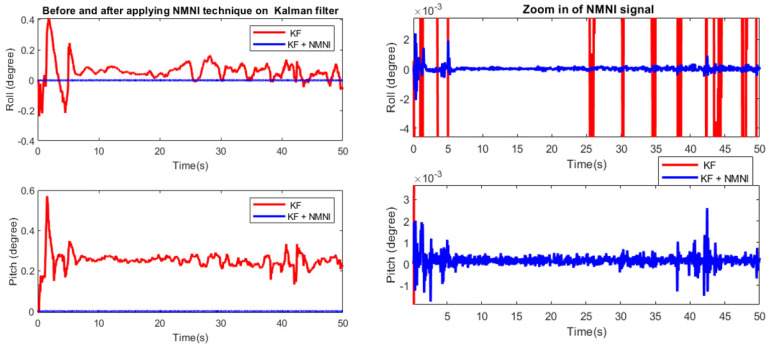
Comparison before and after using NMNI on KF (**left**); zoomed in NMNI signal (**right**).

After the KF accomplishment, the fusion filter was applied to verify the NMNI on KF. Figure 12 shows the error of the KF and the fusion filter, where the error significantly decreased with the function of drift elimination from the NMNI filter. The starting transient in roll and pitch were kept at a balanced state. The KF could not converge the pitch back to the ground truth since the engine vibration continuously impacted it. In Table 2, it was observable that there was still noise in the NMNI combination, but it was already optimized with respect to the single KF. With less variation, the fusion filter could track both roll and pitch with higher stability. 

### 5.2. Yaw Estimation

In this section, the portion of angular velocity was integrated to measure the desired value in the *Z*-axis. Unlike the roll/pitch, the accelerometer could not support the gyroscope for a better output. Therefore, the NMNI technique was employed in the gyroscope for drift optimization. The NMNI threshold was obtained from the previous hovering test, based on the maximum abs. value of ω_Z_, then set as the first threshold for the heading test. 

**Figure 13:**
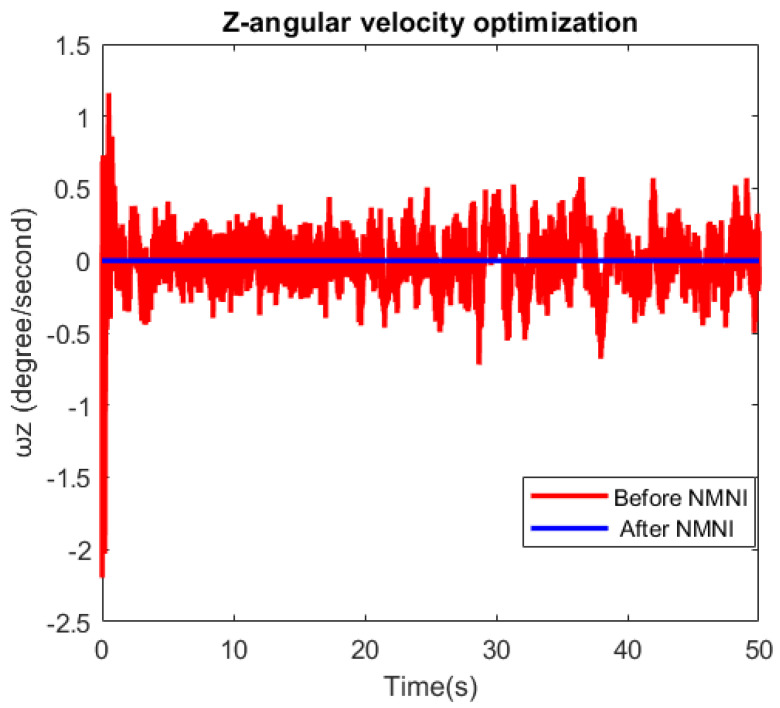
NMNI on Z-angular velocity.

Figure 13 and Table 3 indicate the huge improvement after the NMNI technique, where the value of ω_Z_ stayed around zero at a steady state. The noise and transient were removed in the NMNI signal, as expected. This feature was advantageous since the integration could sum the mini error to become prominent over a time process. Thus, the ω_Z_ was supposed to be as close to zero as possible during the no-motion state. 

**Figure 14:**
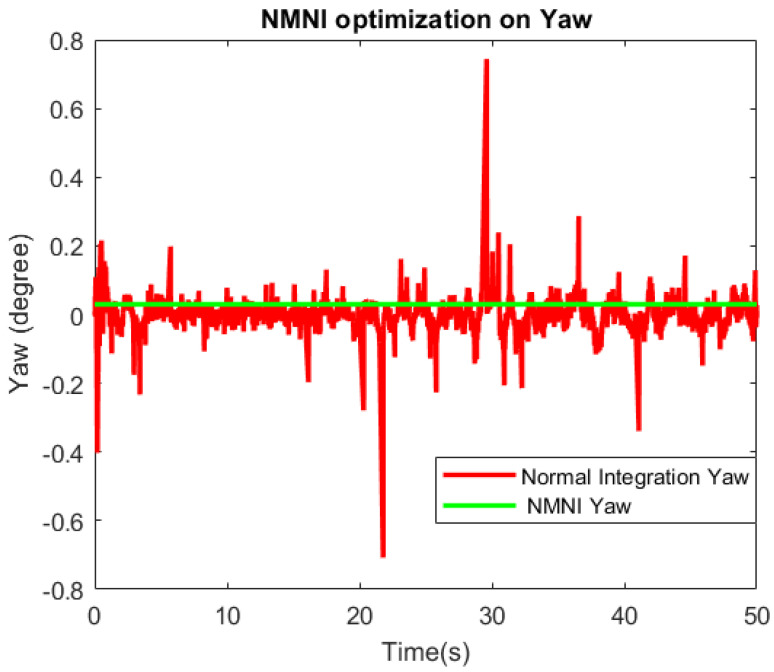
NMNI on yaw behavior.

The yaw performance is evaluated for the heading estimation, as shown in Figure 14. There was a significant difference between the two signals before and after the NMNI filter. The normal integration conducted sudden spikes, which could cause considerable issues for the drone controller. Meanwhile, the NMNI removed these spikes and maintained the signal at the stable state. Furthermore, the absolute error and Std were minimized to track the angle at the proper angle, as reported in Table 4. 

## 6. Conclusions

This research has successfully designed a fusion filter between KF and NMNI, which has accomplished a considerable advancement in the noise optimization of the tilt angle in the application of 6DoF in unmanned aerial vehicles with only an accelerometer and a gyroscope. For the yaw/heading detection, the NMNI, with its updated threshold model, brought a proper measurement by removing the drift during angular velocity integration. 

Generally, mean errors of roll, pitch and yaw are optimally minimized to about 0.02° with low std. This approach relieves the burden of multiple sensors for orientation tracking, reducing the battery energy consumption and helping the drone to obtain the precise angle under engine vibration. In future projects, these algorithms will be improved and expanded to other industrial appliances.

## Figures and Tables

**Table 1:** Before and after KF application.

Abs. Error of Parameters	Before KF		After KF	
	Roll	Pitch	Roll	Pitch
Max. variation (°)	0.53	1.12	0.41	0.54
Mean (°)	0.41	0.53	0.30	0.38

**Table 2:** Sensor fusion comparison.

Abs. Error of Parameters	Roll (°)		Pitch (°)	
	KF	KF + NMNI	KF	KF + NMNI
Mean	0.30	0.03	0.38	0.02
Std	0.06	0.02	0.09	0.01
Max. variation	0.41	0.04	0.54	0.03

**Table 3:** ω_Z_ before and after the NMNI method.

Abs. Error of Parameters	Before NMNI	After NMNI
Mean (°/s)	0.26	0.01
Std (°/s)	0.35	0.01
Max. variation (°/s)	2.08	0.05

**Table 4:** Yaw data before and after the NMNI method.

Abs. Error of Parameters	Before NMNI	After NMNI
Mean (°)	0.15	0.02
Std (°)	0.08	0.01
Max. variation (°)	0.95	0.03
